# Exploring online reproductive health promotion in Canada: a focus on behavioral and environmental influences from a sex and gender perspective

**DOI:** 10.1186/s12889-024-19159-5

**Published:** 2024-06-20

**Authors:** Alexandra R. Rice, Toluwanimi D. Durowaye, Anne T. M. Konkle, Karen P. Phillips

**Affiliations:** 1 Interdisciplinary School of Health Sciences, Faculty of Health Sciences, University of Ottawa, 25 Université Private, Ottawa, ON K1N 6N5 Canada; 2 University of Ottawa Brain and Mind Research Institute, University of Ottawa, Ottawa, ON K1H 8M5 Canada

**Keywords:** Gender, Health promotion, Pregnancy, Health behavior, Environment, Partner, Prenatal care, Canada

## Abstract

**Background:**

Reproductive health promotion can enable early mitigation of behavioral and environmental risk factors associated with adverse pregnancy outcomes, while optimizing health of women + (all genders that can gestate a fetus) and babies. Although the biological and social influences of partners on pregnancy are well established, it is unknown whether online Canadian government reproductive health promotion also targets men and partners throughout the reproductive lifespan.

**Methods:**

Reproductive health promotion, designed for the general public, was assessed in a multi-jurisdictional sample of Canadian government (federal, provincial/territorial, and municipal) and select non-governmental organization (NGO) websites. For each website, information related to environmental and behavioral influences on reproductive health (preconception, pregnancy, postpartum) was evaluated based on comprehensiveness, audience-specificity, and scientific quality.

**Results:**

Government and NGO websites provided sparse reproductive health promotion for partners which was generally limited to preconception behavior topics with little coverage of environmental hazard topics. For women + , environmental and behavioral influences on reproductive health were well promoted for pregnancy, with content gaps for preconception and postpartum stages.

**Conclusion:**

Although it is well established that partners influence pregnancy outcomes and fetal/infant health, Canadian government website promotion of partner-specific environmental and behavioral risks was limited. Most websites across jurisdictions promoted behavioral influences on pregnancy, however gaps were apparent in the provision of health information related to environmental hazards. As all reproductive stages, including preconception and postpartum, may be susceptible to environmental and behavioral influences, online health promotion should use a sex- and gender-lens to address biological contributions to embryo, fetal and infant development, as well as contributions of partners to the physical and social environments of the home.

## Introduction

Reproductive health promotion broadly encompasses interventions that equip individuals with the knowledge, skills, and autonomy to make decisions regarding their sexual, preconception, antenatal and postpartum health [1, 2]. Reproductive health promotion improves pregnancy outcomes, reduces concurrent diseases, and optimizes perinatal outcomes through education and strategies to manage health risk factors [2–5]. Effective reproductive health promotion policies and interventions are inclusive, multisectoral, evidence-based [6], and integrate both sex and gender perspectives into practice [7]. Health promotion for fertility and pregnancy aims to ameliorate reproductive tract disease or dysfunction with emphasis on the biology of sex [1, 2]. By incorporating a gender-based lens, the social, environmental and behavioral influences on reproductive health, including access to health services and autonomy for reproductive decision-making, can be addressed by health promotion [7]. Biological, environmental and psychological modifiers of sex-specific factors relating to gamete quality and the capacity to conceive, gestate and birth a child are particularly relevant during preconception [8] and throughout pregnancy [4, 5]. As gender influences health behaviors, coping strategies, and health services uptake, health promotion interventions tailored to reflect such gendered realities are recommended to redress health inequities [7]. Despite more recent advances to recognize the lived experiences of different genders in pregnancy/parenthood [9], men + (people of all genders who produce sperm) exhibit significant fertility knowledge gaps [10–12], suggested to be related to the exclusion of men + from reproductive health initiatives [12, 13].

Preconception, pregnancy and postpartum stages are ideal for reproductive health promotion as modification of high-risk behaviors and mitigation of environmental exposures can optimize pregnancy outcomes and infant health [3, 5, 14]. Exposures to chemical, physical, and biological environmental hazards may occur through leisure activities, hobbies, home renovations and in workplaces, including healthcare, personal care services, manufacturing, and agriculture [15]. Environmental and occupational exposures may impair sperm quality, with chemicals, solvents, endocrine disrupters, heavy metals and radiation established to induce DNA damage or epigenetic modifications [13, 16–18]. Sperm quality can also be adversely affected by preconception health behaviors including alcohol consumption, use of illicit drugs and marijuana, tobacco use, and poor nutrition practices contributing to obesity [5, 13, 19, 20]. During pregnancy and postpartum, partners, biological/non-biological co-parents and/or co-habitating partners, contribute behavioral influences and other factors which comprise the social environment [13, 21]. Partners can influence the participation of women + in healthy and safe behaviors, prenatal care practices, breastfeeding engagement, and emotional/mental health [13, 21, 22].

Despite the established biological and social influences of partners on perinatal outcomes and infant health, reproductive health promotion has, to date, predominantly targeted women + [2, 4, 5, 13]. Preconception and pregnancy guidelines recommend mitigation of behavioral risks through a healthy diet, folic acid supplementation, regular physical activity, appropriate gestational weight gain, and avoidance of environmental exposures [3–6, 8, 23–26]. Postpartum health promotion focusses on breastfeeding/chestfeeding and parent-infant attachment [22, 24, 25]. Management of behavioral and environmental risks, together with engagement in healthy behaviors, can optimize reproductive health of both women + and their pregnancy outcomes.

Evidence-based, inclusive reproductive health promotion emphasizing modifiable risks to fertility and pregnancy can be effective tools to improve health and pregnancy outcomes [3–6, 27]. About one-third of Canadian women + attend prenatal classes, most commonly primiparous women + , delivered by prenatal educators in hospitals or in community settings [28], and generally hosted by local public health units [26]. Like many prospective parents around the world [29–31], Canadians [32, 33] identify the Internet as the preferred channel of reproductive health information. We have previously reported that Canadian federal and provincial/territorial government agencies provide online promotion of essential prenatal health topics [26], but it is unknown to what extent such reproductive health promotion incorporates a sex- and gender-based lens. We used a multi-jurisdictional approach to evaluate whether Canadian government and select non-government organization (NGO)-hosted websites provide audience-specific (women + , partners) and reproductive stage-specific (preconception, pregnancy, and postpartum) health promotion.

## Methodology

### Sample

Health care in Canada is publicly funded through both federal and provincial/territorial taxation, with multi-jurisdictional responsibilities for priority setting and service delivery. Each of Canada’s ten provinces and three territories, and their respective organization of municipal/regional health authorities, is responsible for delivery of health care services [34]. Canadian government website-hosted reproductive health promotion was evaluated using a multi-jurisdictional approach which included assessment of one federal, all 13 provincial/territorial, and 9 municipal government organization websites [35, 36] (Table 1). Selection of municipal websites emphasized provincial/territorial capital cities and large urban cities. Five Canadian-based, credible NGO websites were purposively selected based on the provision of freely accessible health promotion for individuals of reproductive age, and a stated purpose to provide reproductive and/or parental health content (Table 1).

**Table 1:** Sample of Canadian government-hosted and NGO-hosted websites

Website		Population- 2020 [35, 36]
Federal Government	Government of Canada *(Canada.ca)*	38,027,406
Provincial/Territorial Governments	Alberta *(MyHealth.Alberta.ca)*	4,412,013
	British Columbia *(HealthLink BC, Healthy Families BC)*	5,173,896
	Manitoba *(Healthy Child Manitoba, Manitoba Parent Zone)*	1,381,809
	New Brunswick	783,814
	Newfoundland and Labrador	526,046
	Northwest Territories	44,395
	Nova Scotia	989,154
	Nunavut	39,581
	Ontario *(Ontario Ministry of Children, Community and Social Services, Public Health Ontario)*	14,757,582
	Prince Edward Island	159,179
	Quebec *(Institut national de santé publique du Québec)*	8,551,865
	Saskatchewan	1,165,963
	Yukon	42,109
Municipal Governments	Halifax, Nova Scotia^CMA^	450,910
	Montreal, Quebec^CMA^	4,366,487
	Ottawa-Gatineau, Ontario^CMA^	1,462,582
	Saskatoon, Saskatchewan^CMA^	336,850
	St. John’s, Newfoundland and Labrador^CMA^	213,919
	Toronto, Ontario^CMA^	6,543,886
	Vancouver, British Columbia^CMA^	2,743,765
	Winnipeg, Manitoba^CMA^	850,558
	Whitehorse, Yukon^CA^	33,662
Non-Government Organizations	Best Start	
	The MotHERS Program	
	Ontario Prenatal Education	
	Canadian Mental Health Association	
	Dad Central	

*CMA *census metropolitan area, *CA *census agglomeration

### Data collection

Data was collected between August 2020 and February 2021. Websites were evaluated through (1) general exploration of each website and (2) keyword search. Health promotion topics/keyword search terms (Table 2) were selected based on reproductive health promotion best practices [2–4, 25]. The extracted data corpus included reproductive health recommendations, guidelines, and resources that were publicly available, targeted a lay audience, and employed plain language. Websites hosted by agencies in the Francophone province of Quebec (Government of Quebec, Institut National de Santé Publique du Québec, City of Montreal) were evaluated in both French and English. Ethics approval was not obtained since all health promotion information collected was publicly available.

**Table 2:** Health promotion topics/keywords

*Environmental Health (n=10)*	*Behavioral Factors (n=11)*
Air Quality	Alcohol
Radiation	Cannabis
Workplace Exposures	Tobacco
Secondhand Smoke	Drugs/Medications
Toxoplasmosis	Weight
Bisphenol A (BPA)	Nutrition
Lead	Physical Activity
Mercury	Vitamins
Organic Solvents	Folic Acid
Pesticides	Sexually Transmitted Inflections (STIs)
	Vaccinations

### Evaluation methodology

Evaluation of websites was conducted by a customized evaluation of website health information adapted from literature [37, 38] and informed by qualitative thematic content analysis [39], used previously in the assessment of prenatal guidance documents [6] and online prenatal health promotion [26]. Websites were evaluated individually for selected health promotion topics (Table 2) based on (1) reproductive stage (preconception, pregnancy, postpartum); (2) audience (women + , partners); and (3) scientific quality of information. Assessment of health promotion for each reproductive stage was determined by explicit stage mention or by related terms (e.g. preconception- gametes, sperm, egg, fertility; pregnancy- fetus; postpartum- new parent, neonate/baby, breastfeeding). A sex- and gender-based lens was used to evaluate the biological and social contributions of “partners”- which included biological co-parents (men +) and/or non-biological co-parents of all genders. Audience-specific health promotion related to (1) women + and (2) partners, was scored as 0-the website did not provide information related to the topic, or 1-the website described the topic comprehensively, and included explanation/definition of the topic and relevance to reproductive health. Available health promotion content was further examined to determine scientific quality. Good scientific quality was characterized as health promotion content with reference(s) to government organizations, credible medical associations, and/or scientific literature. Scientific quality was scored as 0-information did not include scientific sources, 1-information included one or more scientific reference(s). Finally, environmental and behavioral ‘breadth scores’ were determined for each jurisdiction. Breadth scores were calculated as the total number of topics promoted by each website for environmental health promotion (described throughout as promotion of the reproductive risks associated with the following environmental hazards: air quality, radiation, workplace exposures, secondhand smoke, toxoplasmosis, BPA, lead, mercury, organic solvents, and pesticides; maximum score 10). Website breadth scores for promotion of behavioral influences on reproductive health (alcohol, cannabis, tobacco use, drugs/medications, weight, nutrition, physical activity, vitamins, folic acid, STIs, and vaccinations; maximum score 11) were similarly calculated. Average breadth scores were then calculated for each jurisdiction and presented as a proportion of the maximum score possible for (1) reproductive stage (preconception, pregnancy, postpartum), and (2) audience (women + , partners). Websites were independently evaluated by two female health sciences researchers (ARR and TDD), with final scores determined by consensus.

## Results

### Promotion of environmental health topics

Health promotion of environmental health topics (Table 2) was evaluated for each website and considered reproductive stage, audience, and scientific quality of information. For partner-specific environmental health promotion, evaluated websites generally targeted the biological co-parent in the preconception period and emphasized biological risks to fertility (Table 3). There was little recognition that environmental hazard exposures not only reduce sperm quality but have the potential to also impair fetal development through epigenetic mechanisms. Workplace exposures, hobbies, and leisure activities of partners may inadvertently increase domestic environmental hazards, thereby contributing to environmental risks to pregnancy, and neonates, however this was not addressed by websites in our sample. Language was typically gender binary, referring to “men/fathers”, and “women/mothers”. Few websites provided comprehensive environmental health promotion for women + during preconception and postpartum, with greater, if inconsistent, promotion of pregnancy-specific environmental health information.

**Table 3:** Environmental health promotion excerpts

***Women* + */pregnancy [radiation]**** “Is it safe to have X-rays while I’m pregnant? Yes, X-rays are generally safe in pregnancy. If your healthcare provider finds you need X-rays for a medical problem or injury, it’s okay to have them. It’s better for your baby that you be healthy. Do all types of X-rays have the same amount of radiation? No. Different types of X-rays have different amounts of radiation. Medical X-rays use very small amounts of radiation. If you’re in need of an X-ray so your healthcare provider can properly treat you, you should have the X-ray."* -Nova Scotia
***Women* + */pregnancy [work exposures, radiation]**** “Most jobs are safe during pregnancy. A few small changes at work can add to your comfort and will help you to have a healthy pregnancy and a healthy baby. Some women must stop working or must change to a different type of work when they are pregnant. Talk to your health care provider about the type of work that you do. You may need to make some changes or take extra care at work while you are pregnant if:* *• You must stand up for long periods of time* *• You must lift, push, or pull heavy items* *• You are in contact with chemicals* *• You work with X-rays* *• You work in a noisy work place* *• Your work place is very hot or very cold…”* -Best Start
***Women* + */pregnancy [secondhand smoke]**** “Pregnant women exposed to cigarette smoke during pregnancy have an increased risk of: Miscarriage. Intrauterine growth restriction. Preterm birth.”* -Ontario Prenatal Education
***Women* + *, partners/preconception, pregnancy [solvents]**** “These compounds found in paint strippers, non-latex paints, plastic adhesives and some dry cleaning chemicals may have adverse effects on fertility and fetal neurodevelopment. Exposure should be avoided at the home and the workplace when planning a pregnancy.”* -Canadian Federal Government
***Women* + */preconception, pregnancy, postpartum [mercury]**** “Most fish and shellfish contain small amounts of mercury that is safe to eat. Large fish, such as marlin and shark that live for a long time and eat other fish can contain higher mercury levels. Eating too much fish that are high in mercury can be harmful, especially for: women who could become pregnant, pregnant and breastfeeding women, children, a fetus and baby are the most sensitive to high levels of mercury, which may lead to problems with learning, walking and talking.”* -Toronto Public Health
***Women* + *, partners/preconception, pregnancy [work exposures, lead, mercury, solvents, pesticides, radiation]:**** “Some workplace conditions may be harmful to: Pregnant women and their unborn child. Reproductive health of both women and men. These conditions can lower the chances of getting pregnant. Ask your health care provider for advice if you are exposed to any of the following chemical, biological and physical hazards in your workplace. In most cases, changes to your work are enough to lower the risks. Chemical Hazards. Heavy metals (lead, mercury, cadmium). Agricultural chemicals such as pesticides and insecticides. Polyhalogenated biphenyls. Organic solvents. Ethylene dibromide and ethylene oxide. Formaldehyde. Make sure you carefully read labels before using or buying products. Physical Hazards. Ionizing radiation (alpha, beta and gamma radiation, x-rays). Excessive noise (may cause hearing loss to either you or your baby).Extremes of either hot or cold. Long work hours. Standing for long periods. Lifting, pulling or carrying. Vibration."* -Ottawa Public Health

#### Environmental health promotion to partners

**Fig. 1:**
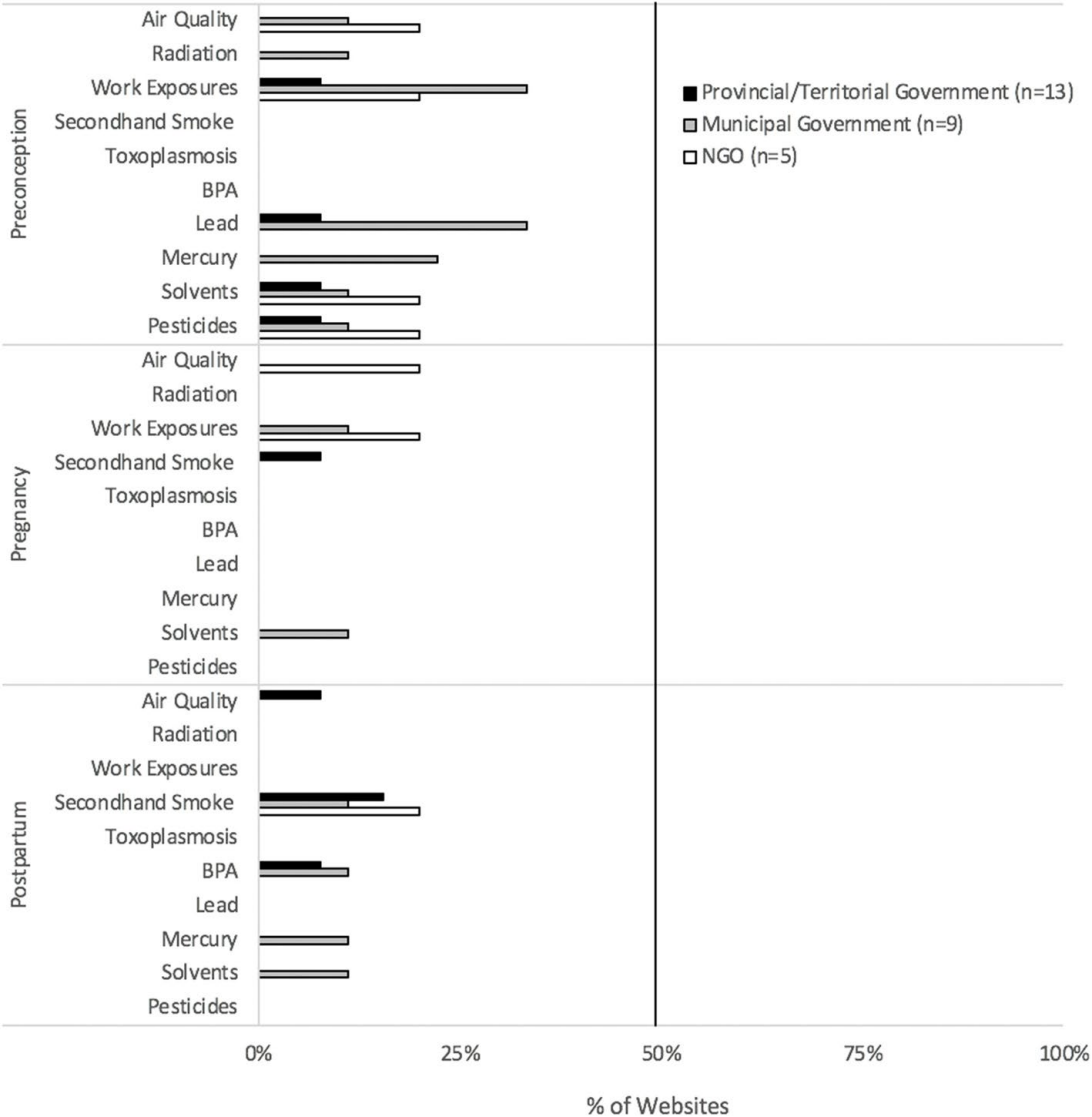
Environmental health promotion for men + /partners. Shown is environmental health information by provincial/territorial government-, municipal government- and NGO-hosted websites for each reproductive stage (preconception, pregnancy, postpartum), targeted to men + /partners. The federal government promoted two (20%) environmental health topics for preconception individuals and no topics for individuals during the pregnancy and postpartum stages. *n* = sample size of websites. BPA-bisphenol A. NGO- non-governmental organizations

**Fig. 2:**
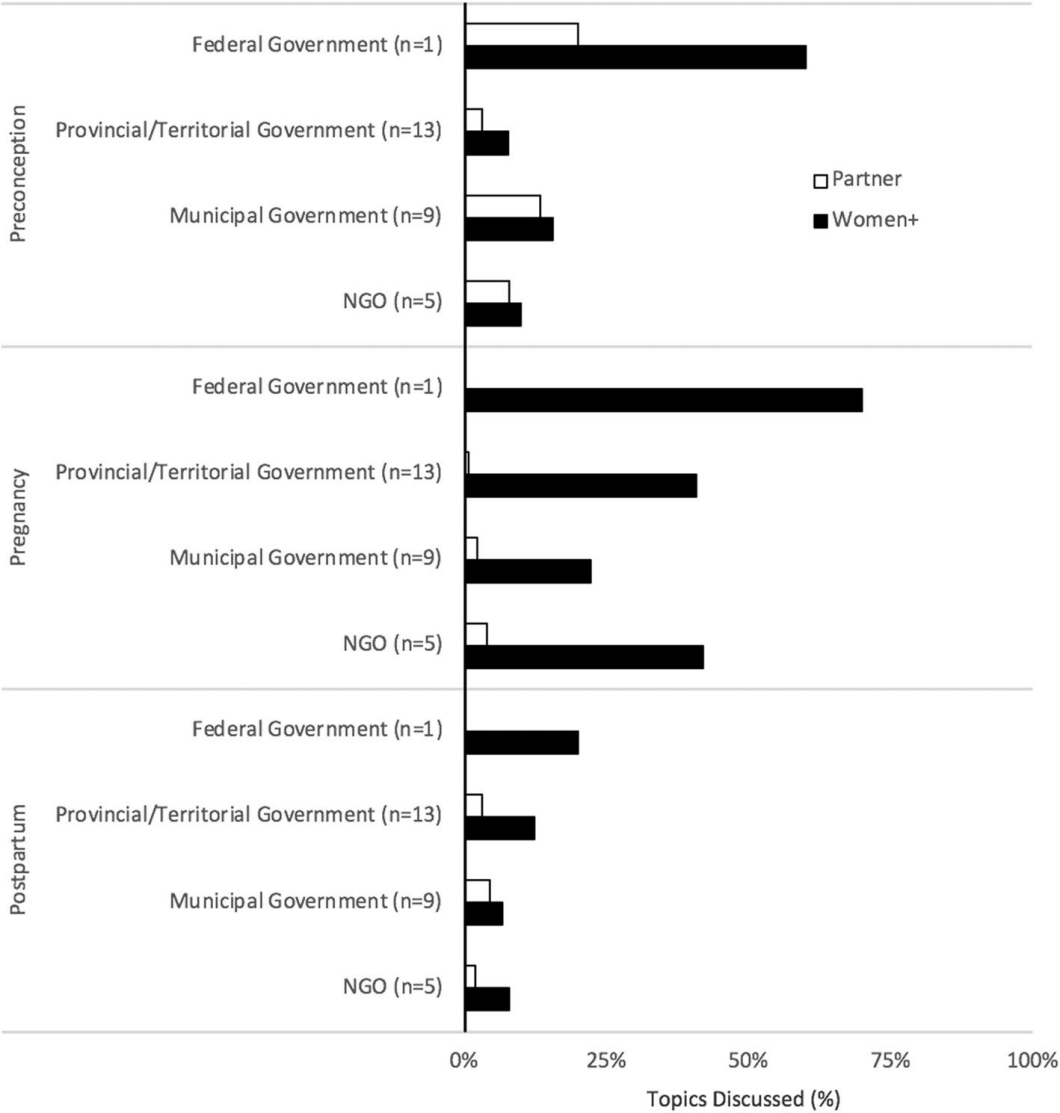
Jurisdictional breadth of environmental health promotion. Breadth scores, the total number of environmental health topics (air quality, radiation, workplace exposures, secondhand smoke, toxoplasmosis, BPA, lead, mercury, organic solvents, and pesticides-maximum score 10) promoted by each website, were averaged for each jurisdiction and presented as a proportion of the maximum score possible. *n* = sample size of websites. NGO- non-governmental organizations

**Fig. 3:**
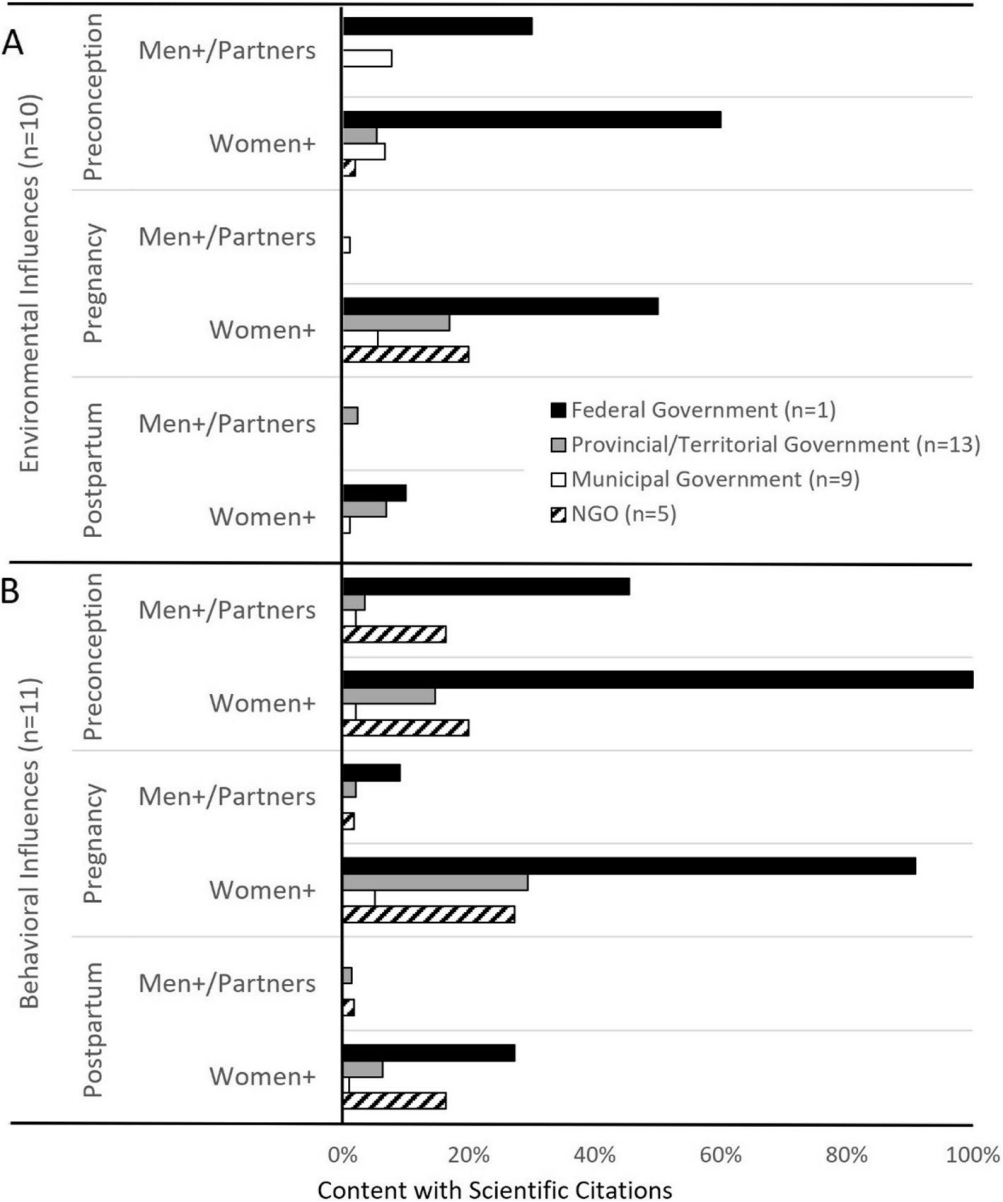
Science quality of health promotion. Scientific quality of reproductive health promotion, targeted to men + /partners or women + , with emphasis on **A**. Environmental health influences, **B**. Behavioral health influences was determined. Health promotion scientific quality is presented as the proportion of website health promotion topics (see Figs. 1 and 2- environment; and Figs. 5 and 6- behavioral) attributed to scientific sources. *n* = sample size of websites. NGO- non-governmental organizations

Partner-specific environmental health promotion was limited for all reproductive stages, particularly in the context of pregnancy and postpartum (Fig. 1). Preconception-related environmental health promotion generally discussed the biological impacts of environmental hazard exposures on fertility. A third (33%) of municipal government websites promoted preconception-related information on lead and workplace exposures. Breadth of environmental health topics for all reproductive stages was limited, especially for pregnancy and postpartum (Fig. 2). Partner-specific environmental health content infrequently included scientific sources (Fig. 3A).

#### Environmental health promotion to women + 

**Fig. 4:**
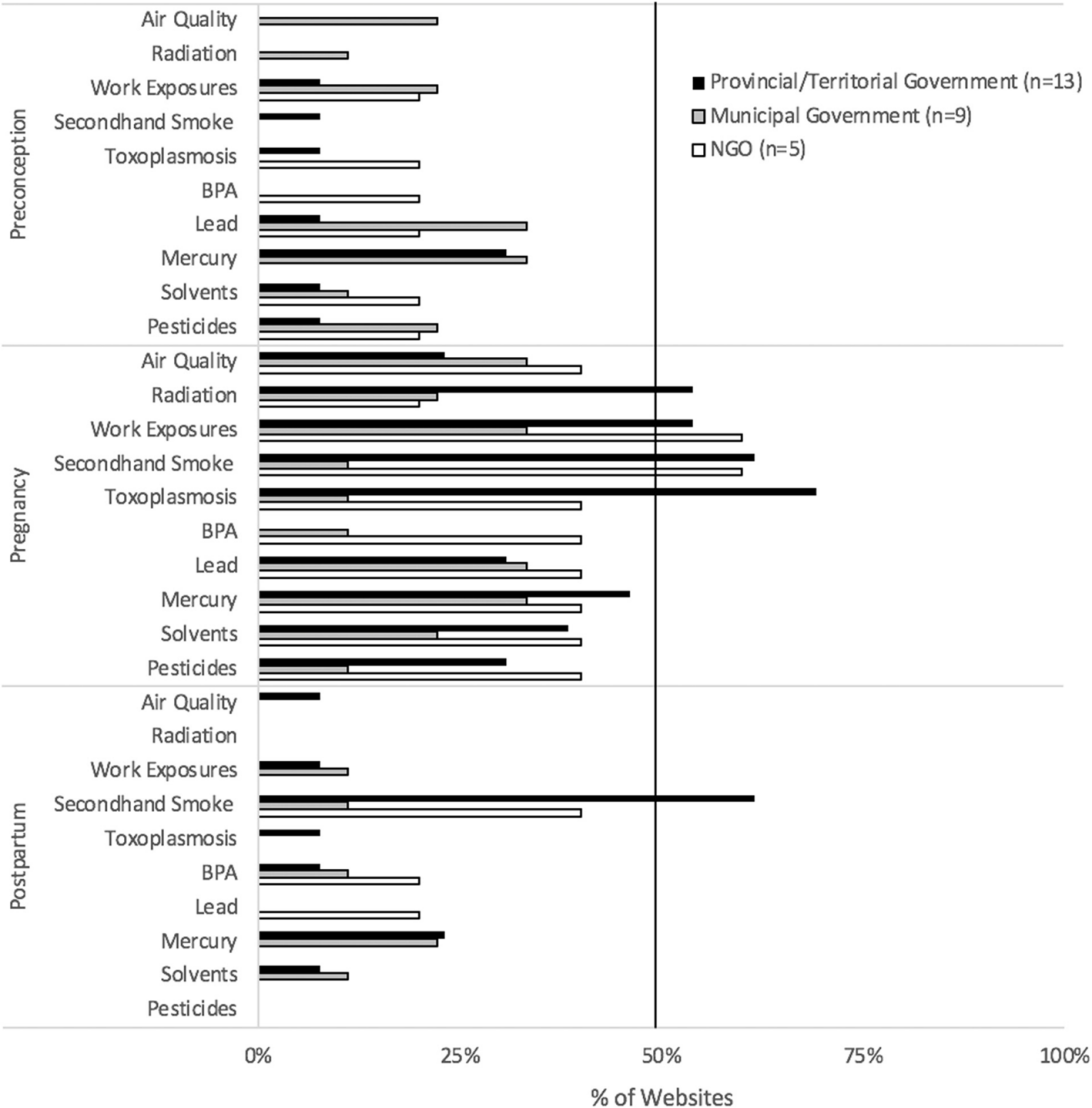
Environmental health promotion for women + . Shown is the environmental health promotion, targeting women + , by provincial/territorial government-, municipal government- and NGO-hosted websites for each reproductive stage (preconception, pregnancy, postpartum). The federal government promoted six (60%) preconception, seven (70%) pregnancy, and two (20%) postpartum-specific environmental health topics *n* = sample size of websites. BPA-bisphenol A. NGO- non-governmental organizations

Environmental health promotion in the context of pregnancy was robust, with several websites within each jurisdiction targeting information to pregnant people (Fig. 4). However, environmental health information for women + during preconception and postpartum was lacking. The federal government promoted 70% of pregnancy-related environmental health topics (Fig. 2). Likewise, over half of provincial/territorial government websites promoted information on radiation, workplace exposures, secondhand smoke, and toxoplasmosis for pregnant people. Over half of NGO websites (60%) provided comprehensive pregnancy-specific information on environmental health in the workplace and secondhand smoke for women + .

For preconception-related environmental health promotion, other than the federal government website which promoted 60% of topics (Fig. 2), no jurisdiction provided significant content breadth. The most common postpartum topic discussed across jurisdictions was secondhand smoke, discussed by 62% of provincial/territorial government and 40% of NGO websites (Fig. 2). Gaps in other postpartum environmental risk topics were evident. Although some environmental health promotion in the context of preconception (60%) and pregnancy (50%) provided by the federal government was attributed to credible scientific/health authorities (Fig. 3A), little environmental health content provided by the other government/NGO websites included citations.

### Promotion of behavioral health topics

We evaluated online health promotion addressing behavioral influences (Table 2) in our sample of websites for each reproductive stage, target audience, noting the scientific quality of information. Websites presented behavioral influences on reproductive health using typically gender-binary language, with content emphasizing women + . Although insufficient, when present, partner-specific behavioral health promotion generally targeted the biological co-parent (i.e. men and fathers) during preconception, with even fewer websites providing pregnancy and postpartum content. Most websites across jurisdictions provided behavioral health promotion targeting women + , emphasizing the biological impacts of behavioral factors on fertility, pregnancy, and fetal/infant development, including breastfeeding as a potential route of exposure (Table 4).

**Table 4:** Behavioral health promotion excerpts

***Partner/preconception [physical activity]**** “Exercise can also help to improve fertility. Moderate physical activity in men has been linked to improved sperm morphology. Exercise might also improve mental health (reducing stress through an increase in endorphins and a decrease in cortisol) and assist with achieving and maintaining a healthy BMI.”* -Ontario Prenatal Education
***Partner/pregnancy [tobacco]**** “If you’re a partner. Keep your home and vehicle smoke-free. If you’re using tobacco, try to cut down and quit. If you’re not ready to quit, smoke or vape outside to support the health of your pregnant partner and baby.”* -Alberta
***Partner/women* + */postpartum [nutrition, physical]**** “You do your best to make sure that your child eats a balanced diet and gets plenty of physical activity. But what about you? You need to take time to exercise and eat healthy meals to stay healthy. All parents need the energy that comes from regular sleep and time-out for yourself.”* -Ontario Prenatal Education
***Women* + */pregnancy [folic acid, vitamins]**** “The incidence of spina bifida in Canada varies by province but is approximately 1/1000 pregnancies. The best way to protect your baby is to start taking folic acid at least 3 months before you get pregnant. This will decrease the chance of your baby developing spina bifida by about 50%. If you wait until you get pregnant or miss your period to start folic acid supplements, you are too late. The spine forms like an open book — and it closes around 42 days from the first day of your last menstrual period, which is approximately 28 days after you ovulate or 14 days after you have missed a period. You only need to take a daily dose of 400 µg, though most vitamin supplements (including prenatal vitamins) contain 1000 µg (1 g). Women who have diabetes or are taking certain epilepsy medications, women who have previously had a baby with spina bifida and women who are obese need to take higher doses of folic acid; ask your health care provider what is right for you”* -MotHERS Program
***Women* + */preconception, pregnancy, postpartum [alcohol]**** “Do not drink alcohol when you're: pregnant or planning to become pregnant, or about to breastfeed… Alcohol affects an unborn baby's development. If you're pregnant or breastfeeding your safest choice is to drink no alcohol at all. Your baby is vulnerable throughout your pregnancy. Any reduction in alcohol consumption at any stage of pregnancy is good.” -Yukon*
***Women* + */postpartum [tobacco]:****“Even if you smoke, breastfeeding is still the healthiest choice for your baby. If you can, try to cut down on smoking or quit. It is best to smoke after you breastfeed your baby. Smoke outdoors while the baby is left inside with family or friends. If you have smoked, wash your hands and change your outer clothing before holding your baby.”* -Best Start
***Women* + */postpartum [alcohol]:****“When you drink, alcohol gets into your breast milk. The amount of alcohol in breast milk depends on how much alcohol you drink. In large amounts, alcohol may affect your baby’s sleep or reduce the amount of milk your baby takes at feeding time. If you are breastfeeding it is safest to limit your alcohol use to one drink or less per day. If you are going to drink alcohol, it is best to feed your baby first, have a drink and then wait two to three hours before you breastfeed again. This allows time for the alcohol to leave your body. A parent who only drink once in a while should still breastfeed, because the benefits are greater than the risks. If you are breastfeeding and plan to have a few drinks, you can express and store your breastmilk ahead of time to give to your baby”* -Manitoba
***Women* + */pregnancy, postpartum [nutrition]:****“Just like in pregnancy, it is advisable for women to eat a variety of healthy foods with an emphasis on whole grains, fruits and vegetable, lean protein, adequate sources of calcium, and good fats. Women should make their calories count and try to limit or avoid eating foods that supply calories with no nutrition, foods that are highly processed, and foods high in salt. With the demands of the new baby, there may not be a lot of time for food preparation. Many frozen or prepared meals can be high in fat and sodium. However, there are stores that sell healthier versions of these meals. Fresh fruits and vegetables can be purchased already cut up and ready to eat. A less expensive alternative is frozen fruits and vegetables that are also high in nutrition. The new parents can suggest that friends and family members bring home-cooked meals and prepared foods on a regular basis. Most new mothers will find snacking and eating smaller meals more time-efficient as they manage to care for themselves and their baby in the first few weeks. Women should be encouraged to drink plenty of water. For more information on nutrition during breastfeeding, refer to the Breastfeeding file.”* -Ontario Prenatal Education

#### Behavioral health promotion to partners

**Fig. 5:**
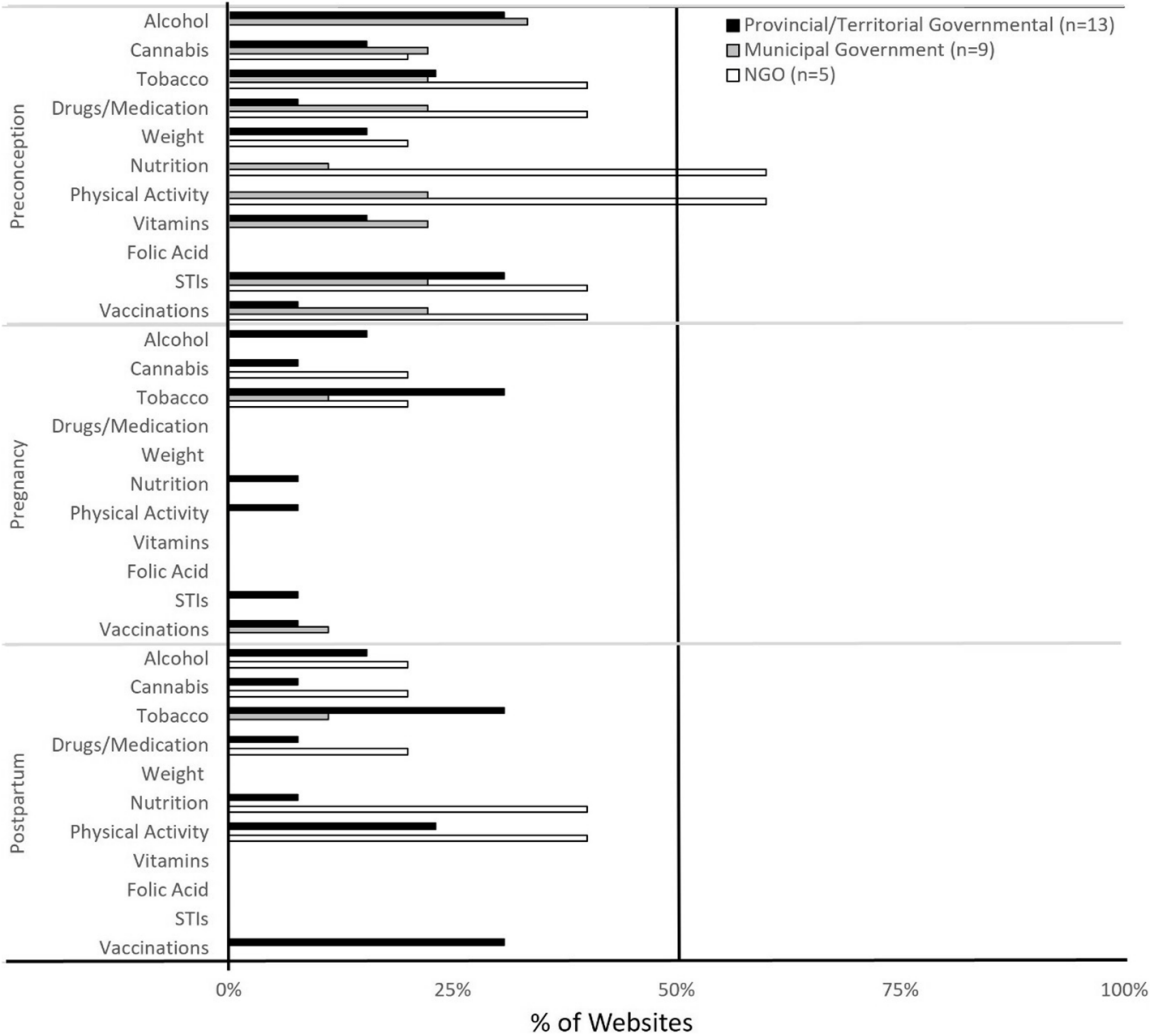
Behavioral health promotion for men + /partners. Shown is the proportion of environmental health information, targeted to men + /partners, provided by provincial/territorial government-, municipal government-l, and NGO-hosted websites for each reproductive stage (preconception, pregnancy, postpartum). The federal government promoted five (45%) preconception, one (9%) pregnancy, and one (9%) postpartum-related environmental health topics. *n* = sample size of websites. STIs- sexually transmitted infections, NGO- non-governmental organizations

**Fig. 6:**
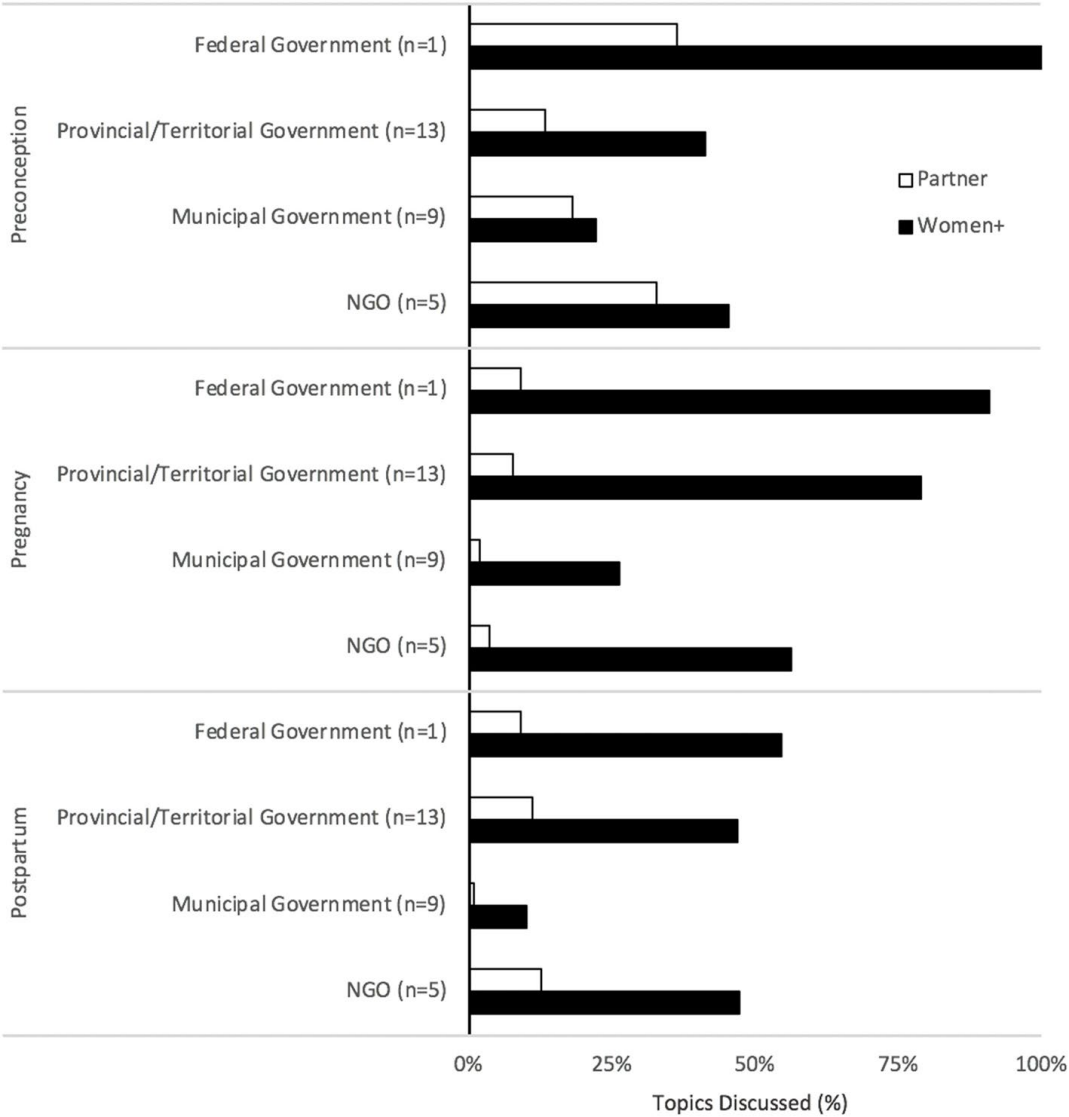
Jurisdictional breadth of behavioral health promotion. Breadth scores, the total number of behavior topics (alcohol, cannabis, tobacco use, drugs/medications, weight, nutrition, physical activity, vitamins, folic acid, sexually transmitted infections, and vaccinations -maximum score 11), promoted by each website, were averaged for each jurisdiction, and presented as a proportion of maximum possible score. *n* = sample size of websites. NGO- non-governmental organizations

For each jurisdiction, less than 40% websites provided behavioral health promotion for partners in the context of preconception (Fig. 5), whereas 60% of NGO websites described benefits of nutrition and physical activity for partner health. Pregnancy-related behavioral health information tailored to partners was particularly limited, with slight improvement, particularly by NGO websites, in the context of postpartum. Partner behavioral health promotion breadth scores were less than 30% for all reproductive stages (Fig. 6), with generally poor scientific referencing across jurisdictions (Fig. 3B), except for the federal government’s provision of partner-specific preconception content, with 45% of topics attributed to scientific sources.

#### Behavioral health promotion to women + 

**Fig. 7:**
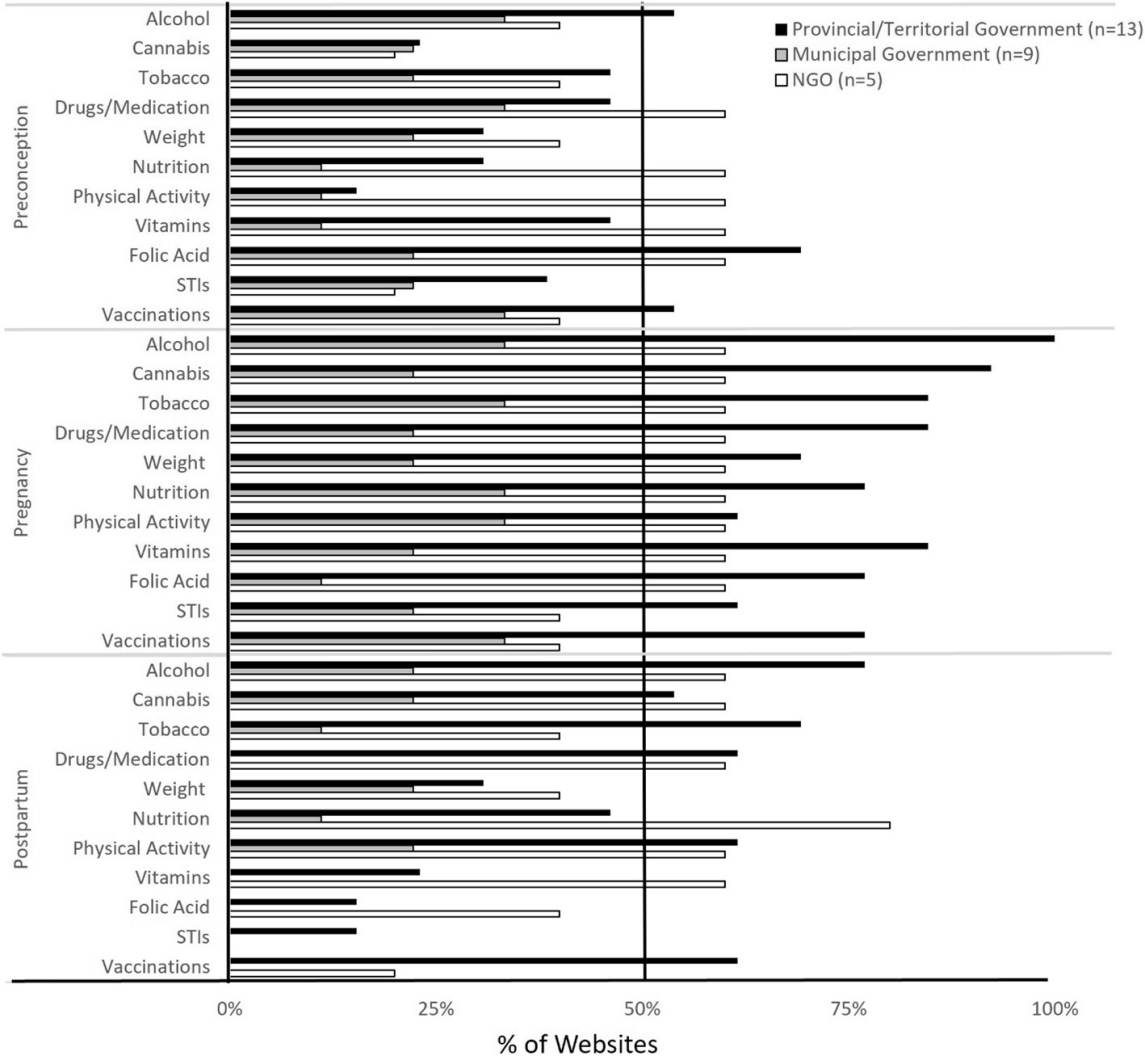
Behavioral health promotion targeting women + . Shown is the proportion of environmental health information, targeting women + , promoted by the provincial/territorial government-, municipal government- and NGO websites for each reproductive stage (preconception, pregnancy, postpartum). The federal government promoted all topics for preconception individuals (100%), and most topics for individuals during pregnancy (91%) and postpartum (55%). *n* = sample size (websites per jurisdiction). STIs- sexually transmitted infections, NGO- non-governmental organizations

Provincial/territorial government websites generally addressed behavioral health promotion targeted to conceiving, pregnant, and postpartum people, in contrast to municipal government websites in our sample (Figs. 6, and 7). Health behaviors during pregnancy were comprehensively discussed by most provincial/territorial government and NGO websites. Over 80% of provincial/territorial government websites promoted pregnancy-specific information on alcohol, cannabis, tobacco use, drugs/medication, and vitamins (Fig. 7). The provincial/territorial government and NGO websites generally provided postpartum-related behavioral health information. Most provincial/territorial government websites (77%) promoted information on alcohol risks, while 80% of NGO websites provided comprehensive information on nutrition for postpartum women + .

In terms of breadth of behavioral health topics, the federal government promoted preconception-specific information on all topics of interest, and most of the topics during pregnancy (Fig. 6). The provincial/territorial government websites exhibited greatest breadth scores during pregnancy, with all jurisdictions dropping below 50% for postpartum content. For scientific quality of behavioral health promotion, in general, the federal government provided scientifically referenced- content, particularly in preconception and pregnancy, otherwise, scientific quality of behavioral health promotion was inconsistent (Fig. 3B).

## Discussion

Canadian government websites generally targeted biological risks of environmental and behavioral factors to women + , typically using gender binary language, with limited partner-specific reproductive health promotion. When present, available partner-specific environmental and behavioral health promotion usually targeted the biological co-parent before conception and emphasized biological risks to fertility. In contrast, robust pregnancy-related environmental and behavioral health promotion was generally provided for women + . Few Canadian government websites presented environmental health promotion during preconception and postpartum stages. Reproductive health promotion targeting women + in the context of preconception and postpartum, emphasized biological risks to fertility, perinatal health, fetal/infant development, and breastfeeding.

### Reproductive health promotion- men + , partners

Biologically, preconception behavioral risks and exposures to environmental hazards pose the greatest threat to sperm quality [8, 13, 16, 17, 19, 20, 21]. In our sample, few websites provided preconception-related reproductive health promotion for partners, with minimal emphasis on environmental hazards. Although most high-risk behavioral behaviors are modifiable, behavioral change requires time, and can be greatly aided by interventions, access to healthcare and other supports [40]. As spermatogenesis cycles are about 74 days, behavioral changes and mitigation strategies to reduce workplace or environmental exposures would need to be implemented several months prior to conception [16, 20]. Further, given that many high-risk behaviors including unsafe sexual practices, and use of recreational drug, alcohol, or tobacco are often concurrent [41], behavioral risks may contribute additively to sperm quality and paternal determinants of fetal development [42].

Whereas the health behaviors of the biological co-parent are important before conception, during pregnancy and postpartum, partners, biological or non-biological parents or co-habitating partners, may provide support, can assist in pregnancy-related decisions, but may also exert behavioral and social control [13]. Partners may influence the perinatal domestic environment through their choices of consumer products, hobbies and behaviors which contribute secondhand tobacco or cannabis smoke, and through workplace-home transfer of occupational chemicals and other exposures [43]. Canadian government websites in our sample generally limited behavioral health promotion to preconception guidance for partners to optimize sperm quality and did not discuss partner contributions and influences during pregnancy and postpartum. Due to the concordant nature of health behaviours among co-habitating partners [44], improved general health practices in partners are important to the health and wellbeing of women + and their offspring. Partners can influence the health practices of the pregnant person and subsequent fetal/infant health and development [13, 44]. Nutrition and behavioral education interventions that involve partners can improve the overall household nutrition knowledge and practices [45, 46]. It is increasingly apparent that health promotion content on nutrition, vitamin supplementation, and physical activity in the context of healthy weight maintenance and pregnancy-related outcomes is beneficial for all members of the household regardless of reproductive stage.

It is well established that men + exhibit gaps in fertility awareness [10–12], partly because reproductive health promotion has historically targeted women + [3, 4, 27]. Strategies to improve baseline reproductive health knowledge, including general fertility and health risk information may begin with a multifaceted approach to reproductive health education. As many unhealthy behaviors established as risks to fertility begin at a young age [23], school-based sexual health education is a strategy to incorporate reproductive anatomy, behavioral and sexual risk reduction [47], consistent with the recommendations for Dutch men + to participate in a fertility awareness campaign [48]. Strategies to encourage men + to have a reproductive life plan, including options for contraception, STI risk reduction, and behavioral risk management can be incorporated in general healthcare visits [13]. Regular engagement with healthcare providers can establish the basis for ongoing reproductive healthcare discussions, including risks, concerns, and options for fertility enhancement or preservation [13]. Inclusion of partners in reproductive life decisions, at the invitation of women + [2], can promote mutual responsibility and strategies for behavioral changes, such as reductions in smoking and alcohol intake [49].

Understanding and evaluating health literacy in men + is recognized as a significant limitation to the health communication field, with overemphasis on the acquisition of information, rather than an understanding of health messaging demonstrated, in part, by changes to behavioral practices [50]. Generally, health promotion targeting men + should use accessible lay language, relate to their everyday experiences, at the same time recognizing the gender and cultural heterogeneity of this population [50]. Uptake of health promotion messaging may be fostered by framing engagement in reproductive health as a positive construct, emblematic of being socially responsible, gender-equitable, and a caring, involved partner, while addressing men’s reproductive-related fears, and concerns [51]. Engaging men + in sexual and reproductive health interventions should be framed using a sexual rights-based approach, that is inclusive, non-discriminatory, promotes autonomy and agency but also challenges stereotypical gender norms [51].

### Reproductive health promotion- women + 

Across our sample of evaluated websites, environmental health promotion targeted to conceiving women + was sparse, with improved promotion of specific topics (radiation, work exposures, secondhand smoke, toxoplasmosis) during pregnancy. As almost half of pregnancies are unplanned [28, 52], ongoing awareness of environmental health risks can empower some individuals to employ the precautionary principle to avoid unnecessary exposures [53], recognizing that not all communities have equal access to environmental justice [14]. Preconception counselling in antenatal care settings can enhance pregnancy-related knowledge, increase awareness, and improve self-efficacy for women + [54], however it remains challenging to increase reproductive health awareness in individuals with no explicit parenthood intentions [27] . Although the most trusted source of health information for Canadian women + is their physician [28], environmental health is recognized as a training gap for most healthcare professionals [55–57], further demonstrating the need for credible, evidence-based environmental health promotion. Ideally, occupational risks should be addressed by occupational health and safety legislation and workplace policies, however implementation of relevant safety measures is often ineffective, requiring employees to assume personal responsibility for the mitigation of reproductive risks [58]. Improving general environmental health literacy may help safeguard reproductive health at work and at home [33, 58], supported by environmental health and safety policies.

Provincial/territorial government and NGO websites promoted alcohol abstinence and folic acid supplementation to women + who are pregnant or attempting to conceive, with most organizations discussing a substantial number of behavioral topics during pregnancy. Preconception behavioral risks including smoking, alcohol consumption, obesity, STI and substance abuse are well established to adversely effect oocyte quality, fallopian tube patency, and epigenetic reprogramming at conception [3–5, 18, 23, 40], as well as teratogenic effects on fetal development [24, 59]. Smoking cessation, for both partners, not only improves gamete quality, but reduces fetal and neonatal exposures to secondhand smoke [60]. Overweight and obesity are recognized risk factors for infertility, perinatal complications, adverse pregnancy outcomes, and are often concurrent with other reproductive risks related to diet and sedentary behaviors [61]. Consistent with established guidelines for physical activity during pregnancy [62], government websites in our analysis promoted physical activity and nutrition through all three reproductive stages. Although pregnancy health promotion often reflected guidelines for appropriate gestational weight gain [63], risks of preconception obesity and interpregnancy weight instability on adverse pregnancy outcomes were not addressed [61]. Multidisciplinary health promotion of weight management, nutrition and physical activity may be beneficial, supported by government messaging, public health, primary healthcare and allied health professionals [52].

The postpartum period is mentally, physically, and emotionally complex, involving physical recovery from pregnancy and the competing demands of life with an infant [64]. Americans [65] and Canadians [28] predominantly give birth in hospitals, attended by obstetricians, resulting in a marked decline in reproductive health promotion to postpartum women + [64, 66]. Online Canadian government postpartum health promotion for women + emphasized breastfeeding transmission risks associated drugs, alcohol, and tobacco consumption, along with mental health and quality of life benefits of nutrition, and physical activity. With the exception of secondhand smoke exposure, our sample of websites provided limited information related to environmental exposures, indicating a significant gap. Environmental health promotion interventions can include strategies to reduce environmental exposures associated with at-home hobbies and consumer products but should also address the take-home pathway- workplace chemical residue on clothing, shoes, and equipment [43].

### Reproductive health promotion best practices

The Internet is a well-established, information channel that can be used to provide discrete and accessible reproductive health information that can mitigate barriers to healthcare access including stigma, resource limitations, childcare and time constraints [29, 33, 47, 67]. Ideally, online reproductive health information is evidence-based, comprehensive, and inclusive [26], however for the lay public, finding credible, evidence-based reproductive health information can be challenging [68], particularly given the explosion of reproductive-related myths and social-media fueled misinformation during the COVID-19 pandemic [69]. Governments collect health surveillance data, develop guidelines and population-based recommendations, and serve as a reference sources for health care professionals [70], supporting reproductive health promotion initiatives. Government websites evaluated here, generally provided credible reproductive health information, though rarely attributed to scientific sources. Content gaps, particularly for men + and partners, prevent these websites from serving as a comprehensive repository of evidence-based reproductive health promotion for the Canadian population. Considerable variability was noted among and between jurisdictions, with the federal government, followed by provincial/territorial government-hosted websites consistently providing a substantial breadth of reproductive health promotion in comparison to the municipal jurisdiction. This may be explained by differences in regional priorities, and the management and financing of local health units/regional health authorities by provincial/territorial governments [34]. Regardless of residency, all Canadians, and indeed global Internet users, can benefit from online reproductive health promotion by government websites, which may be supplemented by information from NGO agencies. Though not reviewed here, the multiple modalities of government reproductive health promotion strategies also include social media, interjurisdictional transfer payments to support regional/local programming- both online and in person- and healthcare delivery [34]. Ultimately, passive consumption of online reproductive health information is best complemented by active engagement in reproductive health programming such as prenatal classes, breastfeeding support groups, and both primary and specialized healthcare, recognizing some groups are traditionally absent from such interventions and require targeted outreach [6]. Such reproductive health programming necessitates local, community service delivery which may involve municipal or regional health units, as well as NGO agencies [47]. NGO agencies and community groups, such as Dad Central, can offer specialized, tailored reproductive health promotion for specific audiences, building on stakeholder involvement and community relationships [6].

Although pregnant and prospective parents are typically avid consumers of reproductive health information, preconceiving individuals who may not have explicit parenthood intentions or concerns about fertility are challenging to target [27]. From a public health perspective, mitigation of the modifiable risks to reproductive health also improves general health outcomes for all genders. Health promotion targeted to specific life stages, from adolescence through the life course, may be recognized as individually relevant and better achieve relevant behavior modifications [27]. Given that many health behaviors are well established years prior to most individuals’ attempts to conceive, early reproductive health promotion through school-based sexual health education would both optimize public health and support healthy pregnancy outcomes [27, 71]. Reproductive health promotion should be clearly framed in the context of optimizing fertility and pregnancy/child health outcomes, and should address topics specific to reproductive health including STI risk reduction, folic acid supplementation, and avoidance of teratogenic exposures, in addition to general public health messaging [4, 71].

Online health promotion has the potential to resonate with specific audiences such as those typically absent from the healthcare system—men + , rural/remote communities, and populations marginalized by racism, colonialism or xenophobia [6, 26]. Although mass health promotion is effective to broadly communicate to the public, ideally health communication messages are relevant, evoke an emotional response, and are tailored to reflect the realities of specific groups [72, 73]. We have previously reported that Canadian provincial/territorial government websites lack specialized prenatal health content for Indigenous, sexually-diverse and immigrant parents [26], although this was not part of the current analysis. We did, however, note that much of the health promotion messaging provided by websites in our sample used gender-binary language, typically women/men, mothers/fathers. In the context of health promotion, gender-binary terms and pronouns may erase the experiences of non-binary, transgender, and gender-diverse individuals as partners, parents, and conceiving/pregnant people [74]. It must also be considered that gender-neutral or ‘desexed’ terminology may obfuscate health promotion messaging, particularly for people with low literacy, limited education, conservative cultural/religious backgrounds, or those belonging to linguistic minority communities [75]. As health promotion aims to educate and increase awareness, reproductive health promotion should strive to balance effective and clear communication of biological sex-based risks, along with social, environmental, and behavioral risks that may be gendered with an inclusive approach that fosters health equity.

Biological sex differences, together with gendered behaviors, coping strategies and uptake of health resources, support the need to target reproductive health communications to specific communities [7, 13]. However, perhaps more importantly, by exclusively targeting reproductive health promotion to women + , structural and social inequities in health and healthcare are perpetuated. A sample of American-hosted preconception care websites, evaluated in 2015, used biomedical language to predominantly emphasize women’s preconception biological risk mitigation [76]. These websites provided only limited content related to men’s contributions to biological and social risks to reproductive health [76], consistent with our assessment of Canadian websites. Gaps in preconception discourse relating to men + not only contribute to reproductive knowledge gaps, but also contribute to the individual and social expectations that place exclusive responsibility for healthy reproduction solely on women + [76, 77]. It is anticipated that by addressing reproductive health knowledge gaps in men + , this will lead to improved participation in preconception care, a greater sense of shared responsibility, and a greater capacity to provide emotional and social support as a partner and parent [13, 77]. Consequently, calls for gender-transformative health promotion require a consideration of gender-based risks to health, moving beyond biological risks [78, 79], and recognition that co-habitating partners, including non-biological parents, may contribute significantly to the social and environmental determinants of pregnancy and postpartum, including infant development.

### Limitations

Our multi-jurisdictional, geographically diverse sampling strategy is a distinct strength of this study. We also strived to recognize the individual contributions of biological (gametes, uterus) and social (concordant health behaviors, reciprocal environmental exposures) determinants of each reproductive stage. An inclusive gender lens was used to recognize the differential contributions of biological and non-biological parents, as well as co-habitating partners. We also acknowledge several important limitations in our study. The evaluated websites cannot be generalized to all reproductive health promotion in Canada. Similarly, websites were evaluated between April 2020 and February 2021 and represent a ‘snapshot’ of reproductive health promotion available online in Canada at that time. Websites evaluated here will continuously improve and update reproductive health information, recommendations, and guidelines as the field evolves. We also acknowledge that the scope of our analysis was limited to government websites and that we did not assess complimentary health promotion channels or programming that may be provided by these organizations such as in-person outreach, antenatal education, mobile applications, commercial advertising, and social media.

## Conclusion

Canadian government websites primarily targeted reproductive health promotion to women + , with emphasis on environmental and behavioral risks to pregnancy. Significant environmental and behavioral information gaps were evident for men + and partners in the context of reproductive health. Online reproductive health promotion is an important resource to complement primary healthcare, community programming and public health interventions. Governments have the capacity and mandate, to design inclusive, evidence-based and comprehensive reproductive health promotion that differentially addresses the needs of women + , men + and partners across the reproductive lifespan.

## Data Availability

All materials are in the public domain at the websites hosted by the organizations indicated in Table 1 of this manuscript.
